# Alteration of 28S rRNA 2′-*O*-methylation by etoposide correlates with decreased SMN phosphorylation and reduced Drosha levels

**DOI:** 10.1242/bio.041848

**Published:** 2019-03-11

**Authors:** Marilyn F. Burke, Douglas M. McLaurin, Madelyn K. Logan, Michael D. Hebert

**Affiliations:** Department of Cell and Molecular Biology, The University of Mississippi Medical Center, Jackson, MS 39216-4505, USA

**Keywords:** rRNA modification, snoRNP, Cajal body, SMN, Drosha

## Abstract

The most common types of modification in human rRNA are pseudouridylation and 2′-*O* ribose methylation. These modifications are performed by small nucleolar ribonucleoproteins (snoRNPs) which contain a guide RNA (snoRNA) that base pairs at specific sites within the rRNA to direct the modification. rRNA modifications can vary, generating ribosome heterogeneity. One possible method that can be used to regulate rRNA modifications is by controlling snoRNP activity. RNA fragments derived from some small Cajal body-specific RNAs (scaRNA 2, 9 and 17) may influence snoRNP activity. Most scaRNAs accumulate in the Cajal body – a subnuclear domain – where they participate in the biogenesis of small nuclear RNPs, but scaRNA 2, 9 and 17 generate nucleolus-enriched fragments of unclear function, and we hypothesize that these fragments form regulatory RNPs that impact snoRNP activity and modulate rRNA modifications. Our previous work has shown that SMN, Drosha and various stresses, including etoposide treatment, may alter regulatory RNP formation. Here we demonstrate that etoposide treatment decreases the phosphorylation of SMN, reduces Drosha levels and increases the 2′-*O*-methylation of two sites within 28S rRNA. These findings further support a role for SMN and Drosha in regulating rRNA modification, possibly by affecting snoRNP or regulatory RNP activity.

## Abstract (precis)

**Summary:** Our findings support a role for SMN and Drosha in regulating rRNA modification, possibly by affecting snoRNP or regulatory RNP activity, and demonstrate that etoposide impacts rRNA modification.

## INTRODUCTION

The major types of modifications in human rRNA are pseudouridylation and 2′-*O* ribose methylation. Human rRNA contains around 100 of each of these modifications, which are performed by small nucleolar ribonucleoproteins (snoRNPs) (Darzacq et al., 2002; Khan and Maden, 1978; Maden et al., 1972; Maden and Salim, 1974; Lafontaine, 2015). SnoRNPs contain a guide RNA (snoRNA) that base pairs at specific sites within the rRNA to direct the modification. There are two kinds of snoRNPs: box H/ACA, which contain dyskerin and are responsible for the pseudouridylation of rRNA, and box C/D, which contain fibrillarin and perform ribosome methylation of rRNA (Kiss, 2004; Baserga et al., 1991; Fatica et al., 2000; Gautier et al., 1997; Schimmang et al., 1989; Szewczak et al., 2002; Tyc and Steitz, 1989; Watkins et al., 1996). Recent work, coupled with advances in the ability to detect pseudouridylation and 2′-*O* methylation modifications in a high throughput format, has shown that rRNA modifications can vary, generating ribosome heterogeneity (Birkedal et al., 2015; Lafontaine, 2015; Incarnato et al., 2017; Krogh et al., 2016; Sharma et al., 2017). The presence of a heterogenous pool of ribosomes may allow for the selective increase of a given ‘type’ of ribosome, leading to specialized ribosomes that are optimized for the translation of certain mRNAs (Lafontaine, 2015). Specialized ribosomes have recently been implicated as a major contributor to tumorigenesis (Marcel et al., 2015, 2013; Truitt and Ruggero, 2016).

One possible method that could be used to regulate rRNA modifications, and hence impact ribosome heterogeneity, is to control snoRNP activity. We have published that RNA fragments derived from some small Cajal body-specific RNAs (scaRNAs) may form regulatory RNPs (regRNPs) that influence snoRNP activity (Burke et al., 2018; Poole et al., 2017). As their name implies, scaRNAs accumulate in the Cajal body (CB), which is a subnuclear domain that takes part in the biogenesis of several different classes of RNPs, including small nuclear RNPs (snRNPs). Like rRNA, the small nuclear RNA (snRNA) component of spliceosomal snRNPs requires pseudouridylation and 2′-*O* ribose methylation modifications for full snRNP functionality (Darzacq et al., 2002; Tycowski et al., 1996; Kiss, 2004; Yu et al., 1998). These modifications in snRNA are guided by the scaRNA component of scaRNPs. (Darzacq et al., 2002; Kiss, 2004). Very interestingly, three scaRNAs (scaRNA 2, 9 and 17) generate nucleolus-enriched fragments of unclear function (Tycowski et al., 2004). We hypothesize that these RNA fragments, and other snoRNAs with uncertain roles, form regulatory RNPs that interact with the snoRNA component of snoRNPs and impact their activity. Therefore, by their interaction with snoRNPs, regRNPs modulate rRNA modifications (Burke et al., 2018; Poole et al., 2017).

Our previous work suggests that proteins enriched in the CB, such as coilin (the CB marker protein), SMN and WRAP53, impact scaRNA 2, 9 and 17 processing (Poole et al., 2016, 2017). SMN is the survival of motor neuron protein, which is mutated in most cases of spinal muscular atrophy and plays important roles in snRNP assembly (Coady and Lorson, 2011; Fischer et al., 1997; Meister et al., 2002; Paushkin et al., 2002; Pellizzoni et al., 1999, 2002). WRAP53 is a scaRNP/telomerase biogenesis factor that interacts with the Cajal body localization signal (CAB box) present in H/ACA scaRNAs and telomerase RNA (Richard et al., 2003; Jády et al., 2004; Mahmoudi et al., 2010; Tycowski et al., 2009; Venteicher et al., 2009; Zhu et al., 2004). In addition to these factors, we reported that Drosha may also contribute to the formation of regulatory RNPs (Logan et al., 2018). Drosha is a member of the RNase III family that initiates microRNA processing (Denli et al., 2004; Lee et al., 2003; Zeng et al., 2005). In the nucleus, Drosha enzymatically cleaves primary-miRNA (pri-miRNA) into the pre-miRNA stem/loop structure that is then transported to the cytoplasm for additional processing by Dicer (Bernstein et al., 2001; Grishok et al., 2001; Hutvagner et al., 2001; Ketting et al., 2001; Knight and Bass, 2001). Reduction of Drosha alters the fragment to full-length ratio of scaRNA 2 and 9, suggesting that scaRNA 2, 9 and 17 may be unorthodox substrates for Drosha (Logan et al., 2018).

Other conditions that may alter scaRNA 2, 9 and 17 processing are various stresses such as that induced by cisplatin or etoposide (Logan et al., 2018). Notably, we observed that the amount of the mgU2-30 fragment derived from ectopically expressed scaRNA9 is significantly reduced in cells treated with etoposide (Logan et al., 2018). In work presented here, we tested if a subset of rRNA modifications are altered by etoposide treatment. We also examined if scaRNA, snoRNA, SMN and Drosha levels were impacted by etoposide. These studies show that etoposide treatment significantly impacts the phosphorylation profile of SMN and reduces SMN interaction with coilin, resulting in gem formation. Etoposide was also shown to increase the 2′-*O*-methylation of 28S rRNA at sites 2388 and 3923, which was also found upon Drosha reduction. Collectively, our results demonstrate that stress conditions can influence rRNA modifications and suggest that these alterations may be mediated by changes in snoRNP or regulatory RNP levels.

## RESULTS

### Increased 28S rRNA A2388 and G3923 2′-*O*-methylation upon etoposide treatment

**Fig. 1.**
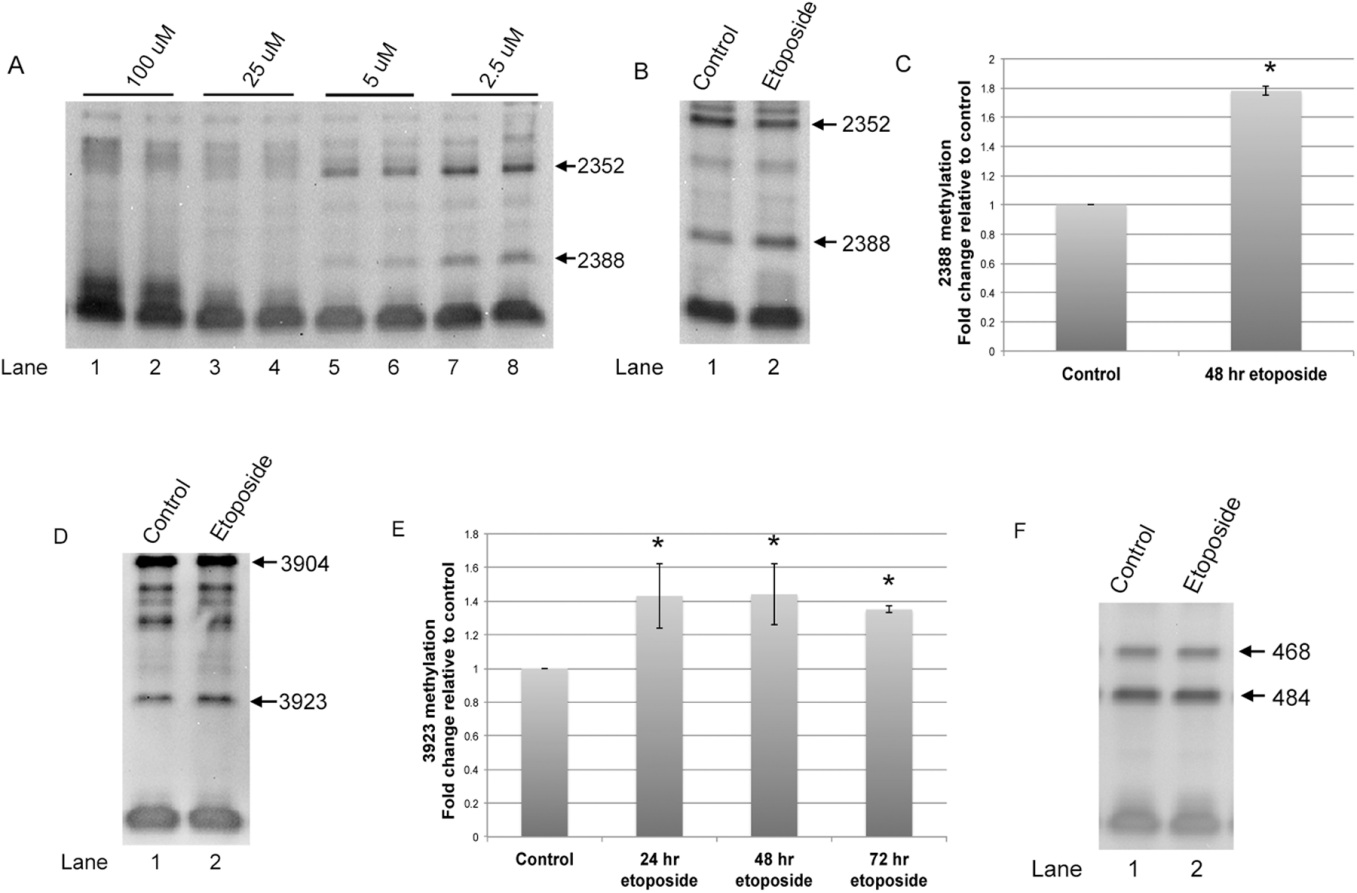
**Etoposide treatment increases the 2′-*O*-methylation of specific sites within 28S rRNA.** (A) Primer extension using untreated RNA and a reducing amount of dNTPs, showing the induction of stop/pause signals corresponding to the 2′-*O*-methylation of 28S rRNA 2388 and 2352 at low (5 μM and 2.5 μM) dNTP concentrations. (B) Low dNTP primer extension using RNA from control or etoposide treated cells to evaluate 2388 methylation. HeLa cells were treated with 9 μM etoposide for 48 h in this experiment. (C) Quantification of 2388 methylation, normalized to the 2352 signal, relative to control, showing an increase in the relative amount of 2388 methylation in response to etoposide (*n*=3 biological repeats, *P<0.05). (D) Low dNTP primer extension using RNA from control or 48 h etoposide treated cells to evaluate 3923 methylation. (E) Quantification of 3923 methylation, normalized to the 3904 signal, relative to control, showing an increase in the relative amount of 3923 signal with 24, 48 and 72 h etoposide treatment (24 h and 72 h, *n*=3 biological repeats; 48 h, *n*=4 biological repeats. *P<0.05). (F) Low dNTP primer extension using RNA from control or 48 h etoposide treated cells to evaluate 18S rRNA 484 methylation. No significant difference was detected (*n*=3 biological repeats).

Our previous results have shown that A2388 and G3923 in 28S rRNA and A484 in 18S rRNA may be subjected to regRNP control (Burke et al., 2018). In addition, we have also found that various stresses, including etoposide treatment, influence the relative amount of fragments derived from scaRNA 2 and 9 that we hypothesize form regRNPs (Logan et al., 2018). To examine if etoposide treatment impacts rRNA ribose methylation, we conducted primer extension assays using reverse transcriptase with low dNTP levels. Low levels of dNTP cause reverse transcriptase to pause near sites of ribose methylation (Maden et al., 1995). Fig. 1A is a representative primer extension assay with decreasing levels of dNTP showing the appearance of stop/pause signals as a consequence of 28S rRNA 2′-*O*-methylation at A2388 and C2352 when dNTP levels are 5 µM or 2.5 µM. RNA from untreated cells or cells treated for 48 h with 9 µM etoposide was then subjected to primer extension with low levels of dNTP to interrogate A2388 methylation. As shown in Fig. 1B, and quantified in Fig. 1C, the amount of 2388 methylation relative to the 2352 signal was significantly increased upon etoposide treatment compared to the untreated control. Primer extension with low dNTP was also used to evaluate 3923 ribose methylation in response to etoposide treatment (Fig. 1D,E). Like 2388, the amount of 3923 signal was increased (relative to 3904) with RNA from 48 h etoposide treated cells compared to RNA from untreated cells (Fig. 1D). This induction in 3923 signal was observed with 24 h, 48 h or 72 h etoposide treatment (Fig. 1E). In contrast, methylation of 18S A484 was not altered by etoposide treatment (Fig. 1F). Collectively, these findings suggest that 2′-*O*-methylation of rRNA is differentially affected by etoposide treatment.

### Induction of scaRNA and snoRNA levels by etoposide

**Fig. 2.**
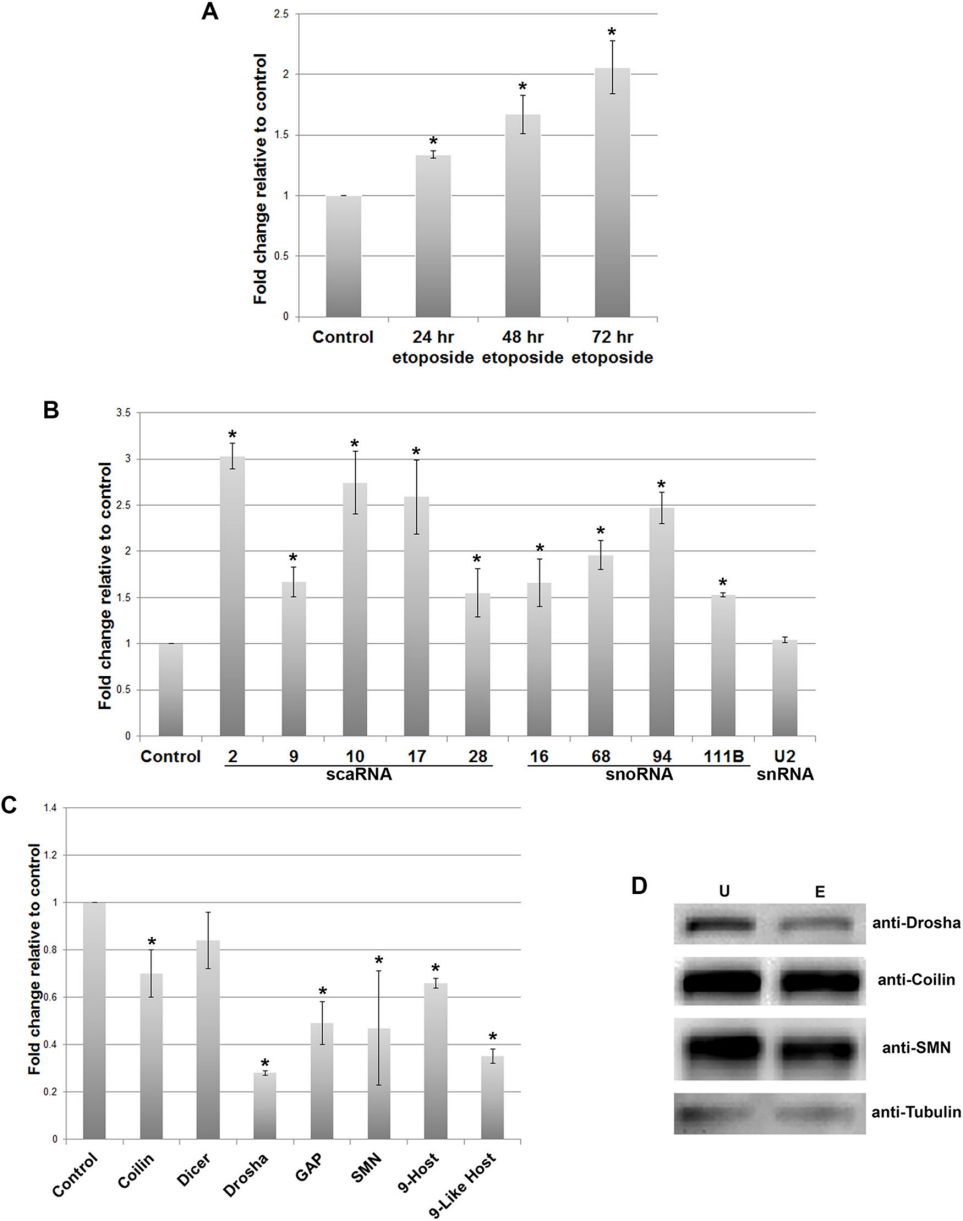
**Etoposide mediated increase of scaRNA and snoRNA.** (A) Reverse transcriptase quantitative real-time PCR analysis of scaRNA9 in RNA from untreated or 24, 48 or 72 h etoposide (9 μM) treated RNA from HeLa cells. 5.8S rRNA was used as the normalizer and data are shown relative to control, which is set as 1 (*n*=3 biological repeats, *P<0.05). (B) Quantification of selected scaRNAs, snoRNAs and U2 snRNA from untreated RNA or RNA isolated from cells treated with etoposide for 48 h. 5.8S rRNA was used as the normalizer (*n*=3 biological repeats, *P<0.05). The data are shown relative to those obtained from untreated control RNA, which is set as 1. (C) Quantification of selected protein coding mRNA, including that from host genes which contain intron-encoded scaRNA9 (9-Host, *CEP295*) and scaRNA9-like (9-Like Host, *EIF1AX*). RNA from untreated control cells or cells exposed to 9 μM etoposide for 48 h was analyzed. 5.8S rRNA was used as the normalizer (*n*=3 biological repeats, *P<0.05). The data are shown relative to those obtained from untreated control RNA, which is set as 1. For A–C, error bars represent standard error about the mean. (D) Western blot of lysate obtained from untreated or 48 h etoposide treated cells. Antibodies to Drosha (top panel), coilin, SMN and beta-tubulin (bottom panel) were used.

The increased 2′-*O*-methylation of 28S rRNA at A2388 and G3923 upon etoposide treatment may be the result of an increase in the snoRNP machinery that conducts these modifications. Additionally, it is also possible that regRNP levels, which are derived from selected scaRNAs, are impacted by etoposide. To indirectly determine the level of these RNPs, we evaluated that amount of the cognate RNA component by RT-qPCR. Using 5.8S rRNA as the normalizer, we first examined the level of scaRNA9 after treatment with etoposide for 24, 48 or 72 h. As shown in Fig. 2A, scaRNA9 is induced at all time points compared to that observed in untreated cells. When evaluating the levels of additional scaRNAs and selected snoRNAs after 48 h etoposide treatment, we found that all of these RNAs were induced relative to that obtained using control RNA from untreated cells (Fig. 2B). Notably, snord68 and snord111B, which are the guide RNAs for A2388 and G3923 2′-*O*-methylation, respectively, are increased by etoposide treatment. In contrast, U2 snRNA levels are not significantly impacted by this etoposide treatment. These findings suggest that the machinery required for the modification of snRNAs and rRNA is increased by this etoposide treatment. Furthermore, increased levels of scaRNA 2, 9 and 17 by etoposide may result in higher levels of regRNPs derived from these scaRNAs compared to that found in untreated cells.

In addition to non-coding RNA, we also examined the level of selected protein coding RNA in cells treated with 9 μM etoposide for 48 h (Fig. 2C). These messages include those mRNAs that produce Coilin, Dicer, Drosha, GAPDH and SMN. Relative to 5.8S rRNA, Coilin, Drosha, GAPDH and SMN mRNA levels are all reduced after etoposide treatment compared to that observed with RNA from untreated cells. Since Coilin, Drosha and SMN message levels were reduced by etoposide, we then examined protein levels from untreated and etoposide treated lysate. As shown in Fig. 2D, only Drosha showed a clear reduction in protein level by etoposide, consistent with the finding that Drosha mRNA was the most reduced of the RNAs we examined (Fig. 2C). Since scaRNA9 and scaRNA9-like are encoded in an intron of the host genes *CEP295* and *EIF1AX*, respectively, we wanted to determine if the increased levels of scaRNA9 observed upon etoposide treatment is a consequence of an increase in the level of the RNA from the host genes. We found that CEP295 (9-Host) and EIF1AX (9-Like Host) mRNA were both reduced by etoposide treatment compared to that observed from control RNA (Fig. 2C). These findings suggest that the increase of scaRNA9 upon etoposide treatment (Fig. 2A,B) is not simply the result of induced host gene transcription.

### SMN is dephosphorylated by etoposide which correlates with gem formation

**Fig. 3.**
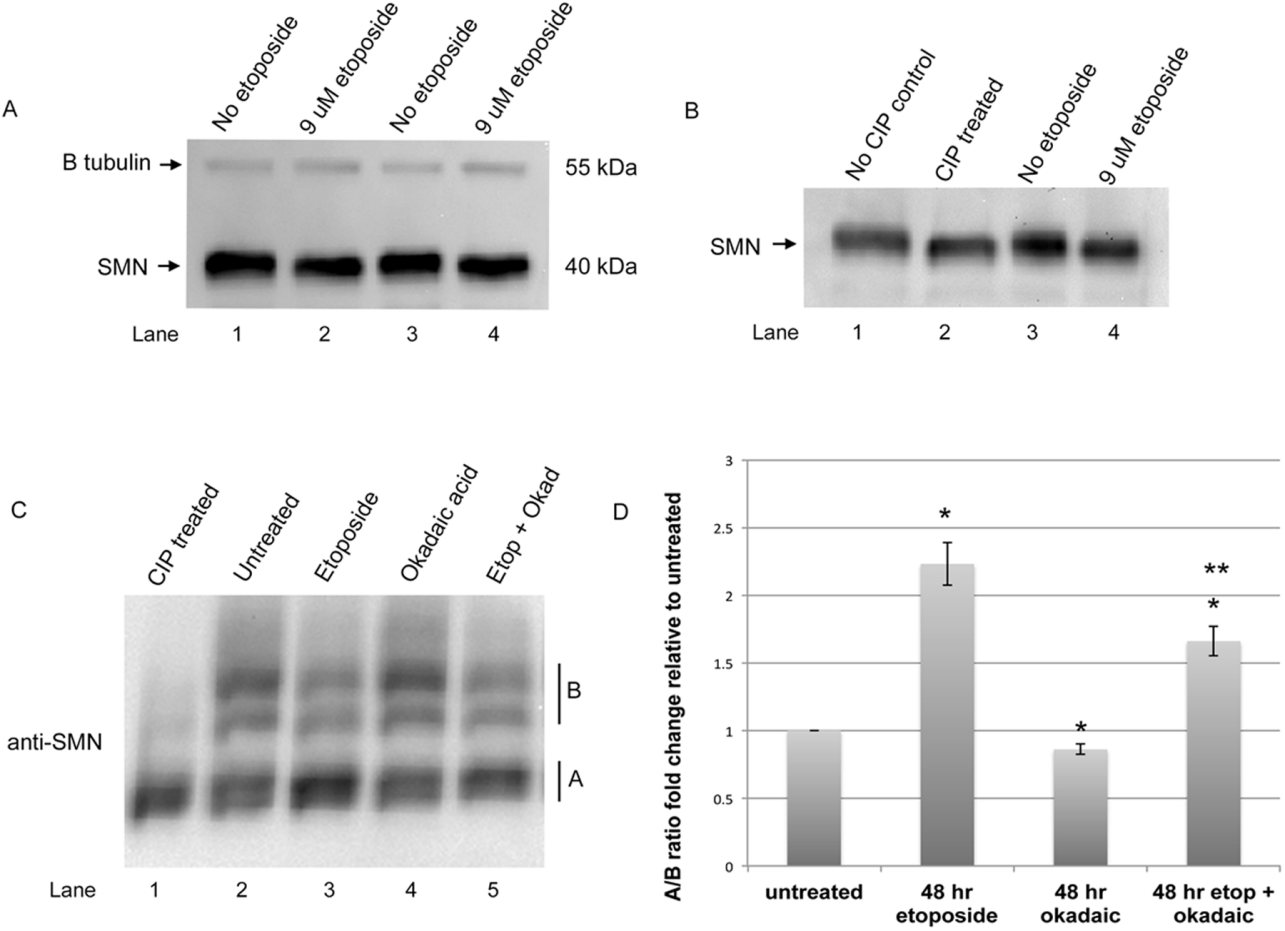
**Hypophosphorylation of SMN by etoposide.** (A) Western blot analysis of lysate from untreated or etoposide treated (9 μM for 48 h) HeLa cells. The blot was probed with antibodies to SMN (bottom) and beta tubulin (top). A slight downward mobility shift is seen in lanes 2 and 4. The estimated molecular weight of SMN (40 kDa) and beta tubulin (55 kDa) is shown. (B) Western blot to detect SMN using lysate treated with alkaline calf intestinal phosphatase (CIP) (lane 2). (C) Migration and detection of SMN using Phos-tag gels, which provide greater resolution of phosphorylated proteins compared to conventional SDS-PAGE. Low or hypophosphorylated SMN is indicated in the A region. More phosphorylated SMN is indicated in the B region. CIP treatment (lane 1) increases the amount of SMN in the A region, consistent with dephosphorylation. (D) Quantification of the signal in the A region divided by the signal in the B region for each condition tested, with the A/B ratio from untreated lysate set to 1. Etoposide treatment increases the amount of SMN in the A region relative to that in the B region by more than twofold compared to lysate from untreated cells (*n*=4 biological repeats, *P<0.05 compared to untreated, **P<0.05 compared to etoposide).

It is known that interactions between coilin and SMN recruit the SMN complex to CBs (Boisvert et al., 2002; Hebert et al., 2001). Specifically, the post-translational modification of coilin by symmetrical dimethylation of arginines within the RG box of coilin mediates association with SMN, and the localization of the SMN complex to CBs. Although we did not observe a large decrease in the amount of SMN from etoposide treated cell lysate compared to control as determined by western blotting, we did detect a slight downward mobility shift (Fig. 3A, compare lanes 1 and 3 to lanes 2 and 4). In contrast, a mobility change was not detected for β-tubulin in etoposide treated cell lysate. (Fig. 3A, upper). We hypothesized that the mobility shift of SMN observed in etoposide treated cell lysate may be the result of decreased phosphorylation. To test this hypothesis, lysate from untreated cells was subjected to calf intestinal alkaline phosphatase (CIP) treatment. SMN in CIP treated lysate migrated more similarly to the SMN from etoposide treated lysate compared to the mobility of SMN from untreated lysate or no CIP control (Fig. 3B). These findings strongly suggest that SMN is hypophosphorylated by etoposide treatment. Because the mobility of SMN is only slightly affected on regular SDS-PAGE by phosphatase or etoposide treatment, we conducted SDS-PAGE using Phos-tag gels which are designed to exacerbate changes in mobility as a consequence of phosphorylation (Wako Chemicals USA, Richmond, VA,). As shown in Fig. 3C, CIP increases the amount of SMN present in a smaller mobility species (denoted by the A region) compared to untreated lysate, which contains larger mobility species that likely contain more phosphorylation (denoted by the B region). Quantification of the low or unphosphorylated SMN (A region) relative to phosphorylated SMN (B region) shows that etoposide treatment increased the relative amount of hypophosphorylated SMN (Fig. 3C,D). Moreover, treatment of cells with the phosphatase inhibitor okadaic acid (Lyon et al., 1997) slightly increased the relative amount of phosphorylated SMN (Fig. 3C,D). In particular, treatment of etoposide treated cells with okadaic acid resulted in a reduction in the amount of dephosphorylated SMN obtained with etoposide treatment alone (Fig. 3C,D). These findings reveal that SMN post-translational modification by phosphorylation is altered by etoposide treatment.

**Fig. 4.**
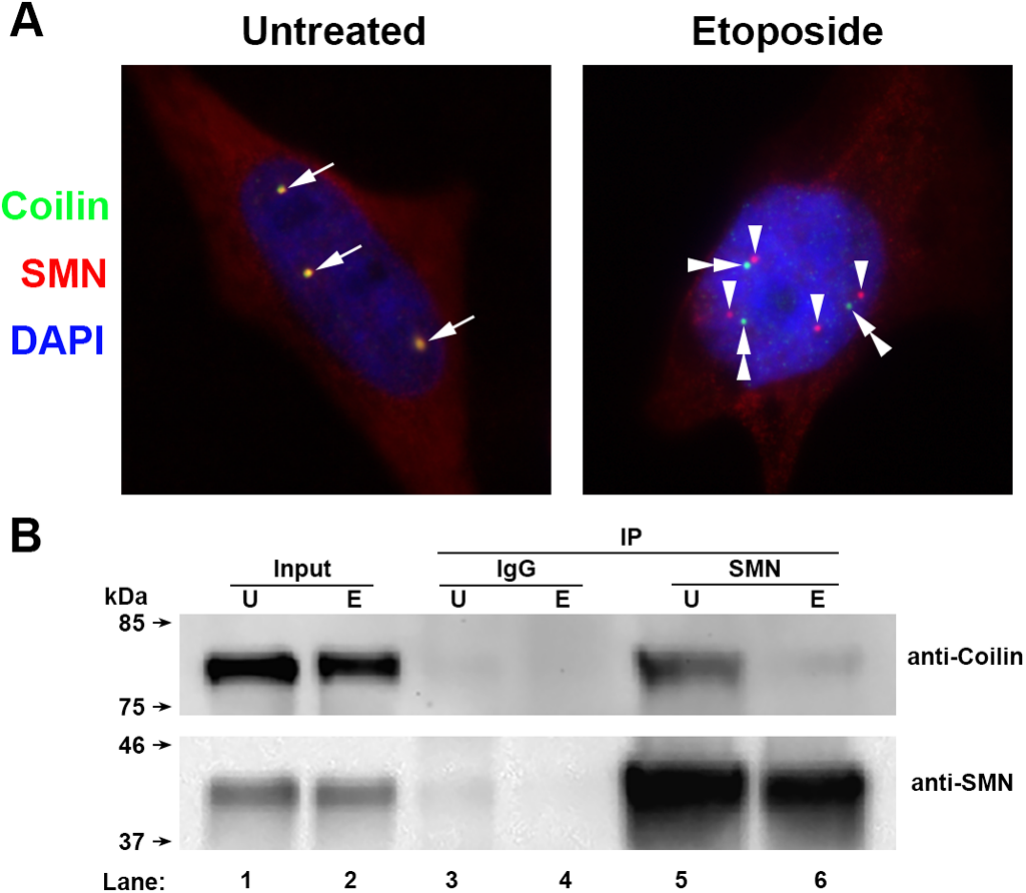
**Etoposide treatment induces gem formation and disrupts SMN interaction with coilin.** HeLa cells were either untreated or treated for 48 h with 9 μM etoposide. The cells were then processed and SMN (red) coilin (green) and nuclei (DAPI, blue) were detected. Arrows indicate co-localization of SMN and coilin in CBs. Arrowheads denote gems, which are SMN foci lacking coilin. Double arrowheads mark coilin foci lacking SMN. (B) Co-IP of coilin by SMN is decreased by etoposide. Untreated or etoposide-treated lysate was subjected to IP with control (IgG, lanes 3 and 4) or SMN (lanes 5 and 6) antibodies. Complexes were recovered by protein G beads, which were then extensively washed, boiled, then run on SDS-PAGE followed by western transfer and detection of coilin (top) or SMN (bottom) using the appropriate antibodies. The input signal represents 2% of that used in the IP reactions.

We have previously reported that etoposide treatment induces the dissociation of SMN from the CB, resulting in gem formation (Logan et al., 2018). For example, HeLa cells incubated with 9 μM etoposide for 48 h have numerous SMN foci lacking coilin (gems) and coilin foci lacking SMN (Fig. 4, right panel, arrowheads and double arrowheads). In contrast, untreated cells have CBs that are enriched for SMN and coilin (Fig. 4, left panel, arrows). To examine if the interaction between SMN and coilin was disrupted by etoposide, leading to gem formation, we conducted co-immunoprecipitation (Co-IP) assays using lysate from untreated or etoposide treated cells. Lysate was incubated with control mouse antibody or a mouse antibody to SMN, followed by capture of complexes on protein G beads, extensive washing, and SDS-PAGE/western blotting. Probing for coilin demonstrates that the amount of coilin Co-IPed by SMN is dramatically decreased in lysate from etoposide treated cells, compared to the amount of coilin recovered when using untreated lysate (Fig. 4B, upper panel, compare coilin signal in lane 6 to that in lane 5). Probing of the same blot for SMN shows that a large amount of SMN was immunoprecipitated from etoposide treated lysate (Fig. 4B, lower panel), and yet the amount of coilin recovered was reduced. These findings support the hypothesis that SMN hypophosphorylation as a consequence of etoposide treatment disrupts the interaction between SMN and coilin, resulting in gem formation.

### Okadaic acid attenuates the increase of 28S rRNA A2388 methylation by etoposide

**Fig. 5.**
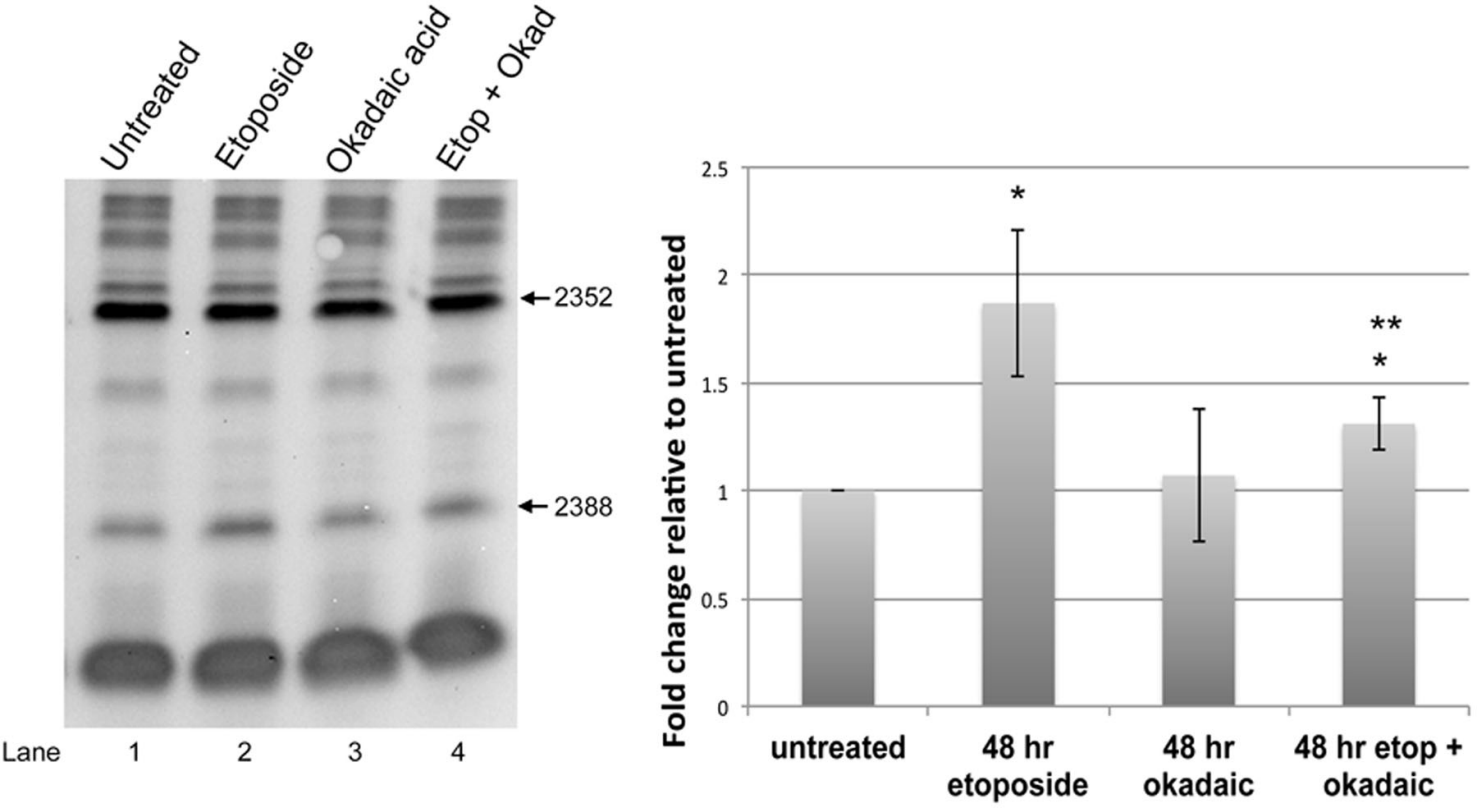
**Etoposide-mediated induction of 28S rRNA 2388 2′-*O*-methylation is reduced by okadaic acid.** (A) Low dNTP primer extension assay to analyze 2388 methylation using RNA from untreated cells or cells treated with 9 µM etoposide, 2 nM okadaic acid, and etoposide (9 µM)+okadaic acid (2 nM) for 48 h. (B) Quantification showing that etoposide+okadaic acid treatment decreases the relative amount of 2388 methylation compared to etoposide alone (*n*=4, *P*<0.05, * compared to untreated, ** compared to etoposide).

Changes in nuclear organization by etoposide may underlie the observed alterations of 28S rRNA A2388 and G3923 2′-*O*-methylation. In particular, etoposide induced gem formation due to hypophosphorylated SMN could disrupt the normal trafficking of snoRNAs and scaRNAs, and possibly impact the formation of regRNPs. To test if we could antagonize the impact of etoposide on A2388 methylation, cells were incubated with the phosphatase inhibitor okadaic acid, alone or in combination with etoposide. RNA isolated from cells with these treatments was subjected to low dNTP primer extension to monitor A2388 methylation. As shown in Fig. 5, RNA from cells treated with okadaic acid and etoposide have significantly less A2388 methylation compared to RNA from cells treated with only etoposide (compare intensity of A2388 band in lane 2 to that in lane 4). Since we have previously shown that SMN phosphorylation is altered by etoposide and okadaic acid treatment (Fig. 3), it is possible that SMN phosphorylation is an important factor in the regulation of specific sites of rRNA 2′-*O*-methylation.

### Okadaic acid increases the relative amount of the mgU2-30 fragment from ectopically expressed scaRNA9

**Fig. 6.**
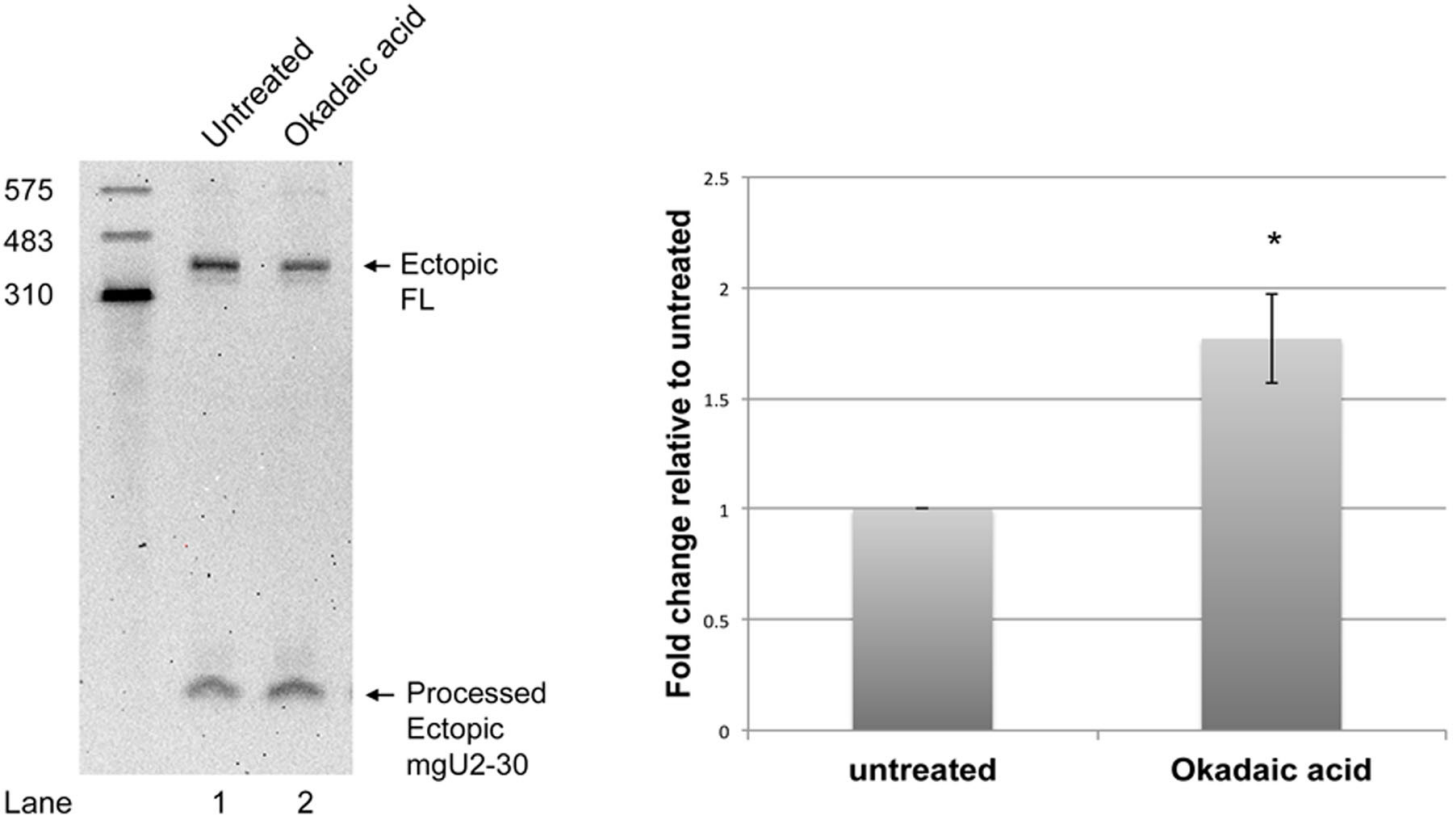
**Okadaic acid alters the dynamics of full-length scaRNA9 and the mgU2-30 fragment.** HeLa cells were transfected with scaRNA9 pcDNA 3.1+ for 24 h. 10 nM okadaic acid was added 7 h after transfection. RNA isolated from untreated and okadaic acid treated cells was then subjected to SDS-PAGE and northern blotting. ScaRNA9 and the mgU2-30 fragment were detected using a DIG labeled probe. Quantification was conducted using these and additional data by dividing the mgU2-30 fragment signal by the full-length scaRNA9 signal for each condition. The mgU2-30/full length scaRNA ratio for untreated cells was then set as 1. Okadaic acid increases the relative amount of the mgU2-30 fragment by approximately 1.7-fold (*n*=4 biological repeats, *P<0.05).

Unlike other scaRNAs, scaRNA 2, 9 and 17 can be processed to generate smaller nucleolus-enriched fragments of unclear function (Tycowski et al., 2004). One of these fragments is mgU2-30, which is derived from scaRNA9. We have proposed that these fragments form regulatory RNPs (regRNPs) that modify the activity of snoRNPs (Burke et al., 2018; Poole et al., 2017). We have also reported that various stress conditions, such as etoposide treatment, alter the ratio of the full-length scaRNA with the derived fragment (Logan et al., 2018). For example, using ectopically expressed scaRNA9, we found that etoposide treatment significantly decreased the amount of the mgU2-30 fragment relative to full-length scaRNA9 (Logan et al., 2018). In addition, etoposide treatment promotes SMN dephosphorylation, decreases coilin interaction and induces gem formation (Figs 3 and 4). Since the phosphatase inhibitor okadaic acid attenuates the etoposide mediated dephosphorylation of SMN (Fig. 3), we next examined if okadaic acid would alter the relative amount of the mgU2-30 fragment derived from scaRNA9. For this experiment, cells were transfected with a plasmid expressing scaRNA9, and treated 7 h later with 10 nM okadaic acid followed by an additional 17 h of incubation. RNA was isolated 24 h after transfection (17 h after okadaic acid treatment) and subjected to northern blotting and detection with a probe that anneals to the mgU2-30 fragment and full-length scaRNA9 (Fig. 6). We observed that, compared to RNA from untreated cells, the relative amount of the mgU2-30 fragment was increased by okadaic acid treatment approximately 1.7-fold. Hence the phosphatase inhibitor okadaic acid and etoposide, which decreases SMN phosphorylation, differentially impact scaRNA9 dynamics in regards to the amount of full-length versus processed fragment.

### Drosha is in a complex with SMN and impacts the 2′-*O*-methylation of A2388 and G3923 in 28S rRNA

**Fig. 7.**
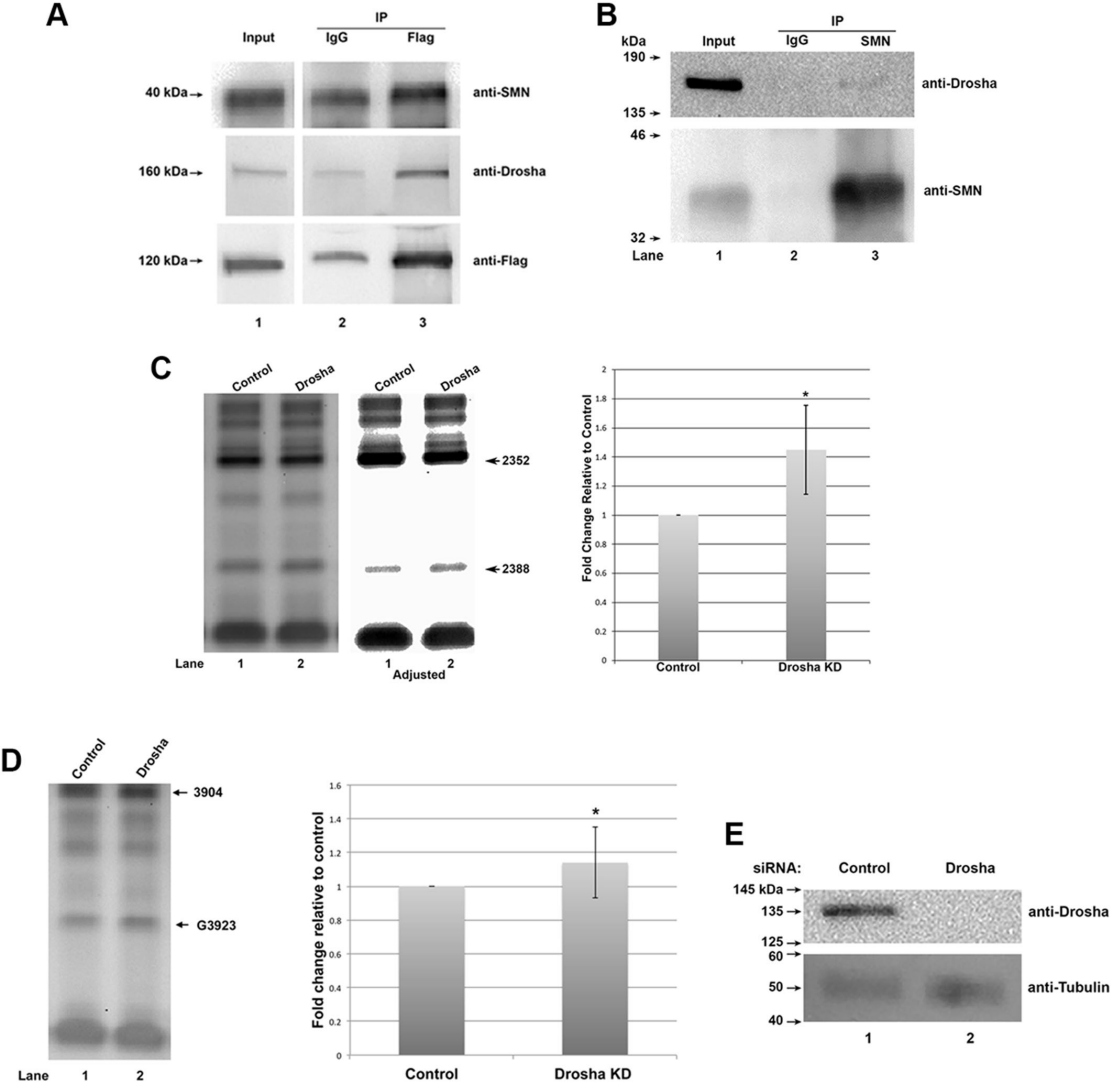
**Drosha interacts with SMN and influences the modification of 28S rRNA A2388 and G3923.** (A) SMN is associated with the Drosha complex. HeLa cells were transfected with FLAG-DGCR8, followed by lysis in KCl lysis buffer and IP with FLAG antibody (Flag) or control mouse antibody (IgG). After complex capture with protein G beads, beads were washed three times with KCl lysis buffer, followed by SDS-PAGE and western transfer. The membrane was probed with antibodies to SMN (top), Drosha (middle) and FLAG (to detect FLAG-DGCR8, bottom). Input represents 4.5% of the lysate used in the IP reactions. (B) Co-IP of endogenous Drosha with SMN. HeLa RIPA lysate was incubated with control antibody or SMN antibody, followed by complex capture with protein G beads. Beads were washed extensively then boiled and run on a SDS-PAGE followed by western transfer and detection of Drosha (top panel) or SMN (bottom panel) using the appropriate antibodies. A faint signal corresponding to endogenous Drosha is seen in lane 3, indicating that SMN and Drosha can form a complex. Reprobing of the same blot with SMN verifies the specificity of the reaction. Input represents 2% of that used in the IP reactions. (C) Low dNTP primer extension to detect 2388 methylation in RNA isolated from control siRNA or Drosha siRNA treated cells. An adjusted image is also shown to more easily visualize the increase in 2388 signal in the Drosha knockdown lane. Quantification was conducted by normalizing the 2388 signal to the 2352 signal and setting the control ratio value as 1. Drosha knockdown increases the relative amount of 2388 methylation by approximately 1.4-fold (*n*=3 biological repeats, *P<0.05). (D) Low dNTP primer extension to detect 3923 methylation in RNA isolated from control siRNA or Drosha siRNA treated cells. Quantification was conducted by normalizing the 3923 signal to the 3904 signal and setting the control ratio value as 1. Drosha knockdown increases the relative amount of 3923 methylation by a very small, but statistically significant amount (*n*=10 biological repeats, *P<0.05). (E) Drosha protein is reduced by Drosha siRNA. A western blot is shown. HeLa cells were transfected with negative control or Drosha siRNA for 48 h. The membrane was probed with Drosha antibody followed by probing with an antibody to β-tubulin.

Our previous results have identified SMN and Drosha as factors that contribute to scaRNA 2, 9 and 17 dynamics (Logan et al., 2018). To examine if these proteins could be in the same complex, we conducted Co-IP experiments using FLAG-tagged DGCR8. DGCR8 is a well-described interactor of Drosha and is part of the Drosha complex. Cells transfected with a plasmid expressing FLAG-DGCR8 were lysed with KCl lysis buffer, followed by sonification and centrifugation. The lysate was then incubated with FLAG antibody (Flag) or control mouse antibody (IgG), followed by complex capture with protein G beads and washing of the beads with KCl lysis buffer. After SDS-PAGE/western transfer, the membrane was probed with antibodies to SMN (Fig. 7A, top panel), Drosha (middle panel) or FLAG (bottom panel). The amount of SMN recovered by the FLAG antibody is more than that recovered by the IgG control. Drosha and FLAG-DGCR8 are likewise found in higher amounts in the FLAG complexes compared to IgG control. These findings show that SMN can associate with the Drosha/DGCR8 complex. We next examined if endogenous SMN could Co-IP endogenous Drosha. For this experiment, cells were lysed in RIPA, which is a more stringent buffer than the KCl lysis buffer used above. RIPA lysate was incubated with control or SMN antibody, followed by complex capture on protein G beads, extensive washing and SDS-PAGE/western transfer. Probing of the blot with anti-Drosha antibodies showed that a faint signal was present in the reaction with anti-SMN but not in the control Ab reaction (Fig. 7B, upper panel, note faint signal for Drosha in lane 3). Re-probing of this same blot with anti-SMN verified that SMN was specifically recovered by the IP reaction containing anti-SMN (bottom panel, lane 3) but not recovered in the reaction with control Ab (lane 2). These findings show that endogenous Drosha and SMN can be found in a complex with one another, and possibly may contribute to scaRNP, regRNP and snoRNP biogenesis. Our previous finding that Drosha reduction alters the dynamics of scaRNA 2 and 9 processing (Logan et al., 2018) supports this hypothesis.

To further implicate Drosha as a factor that impacts rRNA modification, the methylation of 28S rRNA A2388 and G3923 was examined by low dNTP primer extension using RNA from cells treated with control or Drosha siRNA. As shown in Fig. 7C, the methylation of A2388 was significantly increased in RNA from Drosha knockdown cells compared to control knockdown cells. G3923 methylation was also increased with Drosha knockdown (Fig. 7D), but not to the same extent as observed for A2388. Drosha knockdown by Drosha siRNA was verified by western blotting (Fig. 7E). Collectively, these results indicate that Drosha may be a component that helps to regulate rRNA 2′-*O*-methylation.

## DISCUSSION

Ribosomes are not identical, but contain differences, such as variation in ribosomal protein complement and diversity of translation factors, that generate ribosome heterogeneity (Lafontaine, 2015). The major contributor to ribosome heterogeneity is rRNA modification, and the majority of these modifications are snoRNA-guided 2′-*O* methylations and pseudouridylations (Lafontaine, 2015). The ability to detect 2′-*O* methylation and pseudouridylation modifications in a high throughput format has given rise to the hypothesis of ribosome specialization (Birkedal et al., 2015; Sloan et al., 2017; Lafontaine, 2015; Incarnato et al., 2017; Krogh et al., 2016; Sharma et al., 2017). Additionally, increased rRNA 2′-*O* methylation as a consequence of upregulated fibrillarin has been implicated as a contributor to tumorigenesis (Marcel et al., 2015, 2013; Truitt and Ruggero, 2016). Since it is clear that all modification sites within rRNA are not equally modified in a pool of ribosomes (Lafontaine, 2015), a major goal of the rRNA field is to understand how rRNA modifications are regulated.

With this goal in mind, we have designed experiments to examine if a stress (etoposide treatment) known to impact the formation of regRNPs (Logan et al., 2018) disrupts rRNA modification. We also evaluated if etoposide treatment alters SMN and Drosha levels, which are two proteins we hypothesize are involved in the biogenesis of regRNPs (Logan et al., 2018). We have found that the 2′-*O* methylation of two sites within 28S rRNA, A2388 and G3923, are increased upon etoposide treatment but methylation of 18S rRNA A484 is not affected (Fig. 1). Etoposide treatment was also shown to induce selected scaRNA and snoRNA levels, but decrease selected mRNA levels, including that which encodes Drosha (Fig. 2). In addition to Drosha mRNA, Drosha protein levels were reduced by etoposide (Fig. 2D). Thus etoposide treatment increases the methylation of two sites (2388 and 3923) within 28S rRNA and is correlated with reduced Drosha levels. Interestingly, knockdown of Drosha by siRNA was also shown to increase 2388 and 3923 methylation (Fig. 7), supporting a role for Drosha in some capacity as a regulator of rRNA modifications. This hypothesis is further strengthened given that SMN and Drosha can form a complex (Fig. 7A). We are currently conducting *in vitro* studies to directly assess the role of Drosha in scaRNA 2, 9 and 17 processing.

Very interestingly, the phosphorylation of SMN was affected by etoposide treatment. Previous work has shown that SMN phosphorylation influences its localization and SMN complex activity, and the protein phosphatases PPM1G and PP1γ contribute to this process (Aoki et al., 2010; Burnett et al., 2009; Grimmler et al., 2005; Husedzinovic et al., 2015; Husedzinovic et al., 2014; Petri et al., 2007; Poole et al., 2017; Renvoisé et al., 2012). For example, nuclear SMN is hypophosphorylated compared to cytoplasmic SMN given that PPM1G is localized in the nucleus, and this hypophosphorylation is necessary for SMN accumulation in the CB (Petri et al., 2007). In our analysis of SMN protein levels obtained from cells treated with etoposide, we observed a slight downward mobility shift of SMN on standard SDS-PAGE followed by western transfer and detection consistent with dephosphorylation (Fig. 3A,B). Using Phos-tag gels, which have a greater resolution for phosphorylated proteins compared to standard SDS-PAGE, we observed that SMN is indeed more hypophosphorylated upon etoposide treatment compared to control (Fig. 3C). We also observed that the etoposide-induced hypophosphorylation of SMN is attenuated by the addition of the phosphatase inhibitor okadaic acid (Fig. 3C,D). Okadaic acid also blunts the increase of A2388 methylation observed in response to etoposide (Fig. 5) and alters the ratio of the mgU2-30 fragment to full-length scaRNA9 (Fig. 6). These findings suggest that SMN phosphorylation, which is influenced by etoposide and okadaic acid, may impact the regulation of rRNA modification. To more definitively prove the role of SMN phosphorylation in rRNA modification, additional studies utilizing phosphomimic and phosphonull SMN mutants will need to be conducted. Furthermore, the identification of the SMN phosphoresidues that are influenced by etoposide and okadaic acid treatment awaits further investigation.

In regards to nuclear organization, okadaic acid at higher concentrations than that used in our study has been shown to mis-localize CBs to the nucleolus (Lyon et al., 1997; Sleeman et al., 1998), demonstrating that nuclear organization is affected by hyperphosphorylation. We have shown that nuclear organization is also disrupted by etoposide (Gilder et al., 2011; Logan et al., 2018; Poole et al., 2017). Specifically, we have found that etoposide treatment (at 9 μM concentration) induces gem formation (Logan et al., 2018) (Fig. 4). Since etoposide results in SMN dephosphorylation and gem formation, we next tested if the interaction between SMN and coilin was disrupted in etoposide treated cells and observed that it was (Fig. 4B). These results show that SMN phosphorylation is a major contributor to gem formation and coilin interaction, as is the post-translational modification of coilin by symmetrical arginine dimethylation (Boisvert et al., 2002; Hebert et al., 2002, 2001).

Collectively, the data shown here support the hypothesis that various stress conditions which impact regulatory RNP biogenesis may alter rRNA modification. Our data also further strengthen the link implicating SMN and Drosha as contributors towards the generation and regulation of the rRNA modification machinery. Studies such as these will likely continue to reveal novel methods by which non-coding RNAs impact cellular metabolism. For example, a recent study on scaRNA2 demonstrated that this scaRNA promotes chemotherapy resistance by binding miR-342-3p (Zhang et al., 2018). It is probable that non-coding RNAs in the nucleolus packaged in regRNPs likewise interact with snoRNAs and thereby regulate snoRNP activity, resulting in ribosome heterogeneity.

## MATERIALS AND METHODS

### Cell lines, cell culture, plasmid, transfection and drug treatments

HeLa cells were obtained from the American Type Culture Collection (Manassas, VA, USA) and were cultured in DMEM media (Invitrogen) supplemented with 10% heat inactivated fetal bovine serum (Gibco) and 1% penicillin streptomycin (Corning, Manassas, VA, USA). Cells were cultured in a 5% CO_2_ incubator at 37°C. Sca9 was ectopically expressed using the pcDNA3.1+ expression vector as previously described (Enwerem et al., 2014, 2015; Poole et al., 2017). FLAG-DGCR8 plasmid was obtained from Addgene (Watertown, MA, USA). For transfection of 60 mm dishes, 1 μg of plasmid was diluted in 97 μl Opti-MEM (Gibco) and 3 μl Fugene HD (Promega) was added and allowed to complex for 5 min before adding to cell culture. For experiments with drug treatments, cells were seeded a day in advance to be 70–100% confluent at time of treatment. Etoposide (Toposar, Teva Parenteral Medicines, Inc, Irvine, CA, USA) at 9 μM or Okadaic acid at 2 or 10 nM was added, depending on experiment, for 17, 24, 48 or 72 h. For siRNA transfections, RNAiMax was utilized (Invitrogen), and siRNAs are as described previously (Logan et al., 2018).

### Quantitative real-time PCR

RNA was extracted from 48 h transfected HeLa cells with TRI-REAGENT (Cincinnati, OH, USA) according to the manufacturer's suggested protocol. Reactions were set up with 50 ng total RNA in Brilliant II SYBR Green qRT-PCR master mix (Agilent, Santa Clara, CA, USA) using an Agilent MX3000P qRT-PCR system. Amplification rates, Ct values and dissociation curve analyses of products were determined using MxPro (version 4.01) software. Relative expression was determined using the 2^−ΔΔCT^ method (Livak and Schmittgen, 2001). Microsoft Excel was used for post-hoc statistical analysis using the Student's *t*-test. Oligonucleotides used were obtained from Integrated DNA Technologies (Coralville, Iowa, USA) and were as follows:

GAPDH forward (5′-GACTCATGACCACAGTCCATGCCATC-3′), reverse (5′-GACTCATGACCACAGTCCATGCCATC-3′),

ScaRNA2 forward (5′-CGTGTTAGGCGAGTGCGTGCGCCCACC-3′), reverse (5′-ATCAGAATCGCCTCGATAATCA-3′),

scaRNA9 forward (5′-GGGCAATGATGAAAAGGTTTTACTACTGATCTTTG-3′), reverse (5′-TGAGCTCAGGTCAAGTGTAGAAACCATC-3′),

scaRNA9 host forward (5′- TTAAGCTGAAGGAATCTGTTGTTGAA-3′), reverse (5′-CTTATCATCTGGCTTCACAGTTGGAC-3′),

scaRNA9-like host forward (5′- GTAAAGGAGGTAAAAACAGACGCAG-3′), reverse (5′- CTGACCATCCTCTTTGAATACCAGTTC-3′),

scaRNA10 forward (5′-GCCACATGATGATATCAAGGCTG-3′), reverse (5′-GCCATCAGATTACCAAAGATCTGTG-3′),

ScaRNA17 forward (5′-GCTGGACCCGGACCGGTTTTGGG-3′), reverse (5′-AAGGAAAATACTGCGGGCTCATCC-3′),

ScaRNA28 forward (5′-GCAAAGTGATGAGTAATACTGGC-3′), reverse (5′-GCAATCAGATCTTATCAGTTTG-3′),

snord16 forward (5′- TGCAATGATGTCGTAATTTGCG-3′), reverse (5′-TTGCTCAGTAAGAATTTTCGTC-3′),

snord68 forward (5′-CGTGATGACATTCTCCGGAATC-3′), reverse (5′-AAATGTGCTTTCATCAAGGCCG-3′),

snord94 forward (5′- CAGGCTGTGATGATTGGCGCAG-3′), reverse (5′-CAGGCTCAGATTGAGGCAACAG-3′),

snord111B forward (5′-TGTTTTCATCAGCCTGAAGTG-3′), reverse (5′-GAGGCCTGATCAGACACACA-3′),

U2snRNA forward (5′-TTTGGCTAAGATCAAGTGTAGTATCTGTTC-3′), reverse (5′-CTGCTCCAAAAATCCATTTAATAT-3′),

5.8S rRNA forward (5′- CGGCTCGTGCGTCGAT-3′), reverse (5′-CCGCAAGTGCGTTCGAA-3′),

Coilin forward (5′-CTTGAGAGAACCTGGGAAATTTG-3′), reverse (5′-GTCTGGGGTCAATCAACTCTTTCC-3′),

Dicer forward (5′-GGTGGTTCGTTTTGATTTGCC-3′), reverse (5′-GGCAGTGTTGATTGTGACTC-3′),

Drosha forward (5′-GAGACCTAGCCTAGTTTTCCTG-3′), reverse (5′-AATGCACATTCACCAAAGTCAA-3′),

SMN forward (5′-GTG GTT TAC ACT GGA TAT GGA AAT AG-3′), reverse (5′-GAT TTA TTT CCA GGA GAC CTG GAG TTC-3′).

### Primer extension assay to detect 2′-*O*-methylation of RNA

RNA was extracted from 24, 48 or 72 h treated or transfected HeLa cells with TRI-REAGENT (Cincinnati, OH, USA) according to the manufacturer's suggested protocol. 2 μg RNA was prepared with 1 μl Reverse Transcriptase buffer (New England Biolabs, Ipswich, MA, USA), 1 μl of 5 μM dig labeled primer designed to base pair downstream of the methylation site of interest (Integrated DNA Technologies, Coralville, Iowa, USA) and DEPC H_2_O to 8 μl. After 2 min at 95°C and 10 min at 42°C, 1 μl Reverse Transcriptase (New England Biolabs, Ipswich, MA, USA) and 1 μl dNTPs were added and samples returned to 42°C for 1 h. The amount of dNTPs used are as noted, or were low concentrations (2.5 μM or 5 μM) used to detect ribose methylation. Samples plus loading buffer were run on a pre-warmed 15% TBE urea gel (Invitrogen) in 1× TBE at 180 V for 80 min. Gel was then rinsed in 1× TBE for 10 min. cDNA product was transferred to membrane using iBlot DNA transfer stacks (Invitrogen) with the iBlot Gel Transfer device (Life Technologies) using program 5 for 3 min, rinsed in ultrapure H_2_O and crosslinked at 120 K μJ/cm^2^. Membrane was incubated in Roche 1× blocking buffer for 15 min with slow rotation, then 30 min with slow rotation in Roche Anti-Digoxigenin-AP Fab fragments at 1:10,000 in Roche blocking buffer and washed with slow rotation in 1× wash buffer, (Roche wash and block buffer set, Roche, Mannheim, Germany). Membrane was developed with 1× CSPD in development buffer at 1:100 for 5 min at room temp, then placed between transparencies for 15 min in a 37°C incubator. Chemiluminescent images were captured and quantified with a Bio-Rad Chemi Doc Universal Hood and Quantity One Software (Bio-Rad, Hercules, CA, USA). Digoxigenin labeled DNA oligonucleotides were obtained from Integrated DNA Technologies (Coralville, Iowa, USA) and are as follows: 18S rRNA A484 site; 5′-DiGN/GCGCGCCTGCTGCCTTCCTTGGA-3′, 28S rRNA G3923 site; 5′-DiGN/CGCCGGGGGCCTCCCACTTATT-3′, 28S rRNA A2388 site; 5′-DiGN/CCCATGTTCAACTGCTGTTCAC-3′.

### Western blotting and Co-IP

HeLa cells were lysed in RIPA buffer (50 mM Tris HCl pH 7.6, 150 mM NaCl, 1% NP-40, 0.25% Na-Deoxycholate, 1 mM EDTA, 0.1% SDS) plus Protease Inhibitor Cocktail (Thermo Fisher Scientific) and placed on ice. Cultures were collected into microtubes and sonicated briefly before centrifugation for 15 min at 4°C, 12,000 RPM. 10–15 μl of samples were run on SDS page using precast Bio-Rad 10% gels (Bio-Rad). For Co-IP experiments, lysate was incubated with 4 µg SMN or control mouse antibody for 1 h, followed by the addition of a protein G slurry and subsequent overnight incubation with mild shaking. The bead complexes were then washed five times with 1.5 ml per wash with RIPA plus Protease Inhibitor Cocktail, re-suspend in SDS-PAGE loading buffer, boiled, centrifuged and subjected to SDS-PAGE. Gels were run at 200 V for 55–60 min. Where noted, cells were lysed in KCL lysis buffer (20 mM Tris, pH 8.0, 100 mM KCl, 0.2 mM EDTA) followed by sonication and centrifugation as described above. Lysate was subjected to IP with 3 μg FLAG antibody or control mouse antibody. For these reactions, beads were washed three times with KCL lysis buffer before the addition of SDS-PAGE loading buffer. For Phos-tag gels, 7.5% precast zinc containing Phos-tag gels were obtained from Wako Chemical (Wako Chemicals USA, Richmond, VA). HeLa cells were lysed in RIPA buffer without EDTA. Phos-tag gels were electrophoresed in a cold room at 200 V for 55–60 min, followed by two 10 min rinses in 1× transfer buffer containing 10 mmol EDTA with gentle rotation and an additional 10 min rinse in transfer buffer alone. Transfer and detection of western blots were described previously (Poole et al., 2016). A Chemidoc system (Bio-Rad) was used to image the blots and adjustments to images were made using the transformation settings on QuantityOne software and applied across the entire image.

Antibodies used include: SMN, mouse/monoclonal (610646), BD Transduction Laboratories (San Jose, CA, USA); Beta tubulin, mouse/monoclonal (T5201), Sigma-Aldrich; Drosha, rabbit/monoclonal (D28B1), Cell Signaling; Coilin, rabbit/polyclonal (sc-32860), Santa Cruz Biotechnology; Control mouse IgG (sc-2025), Santa Cruz Biotechnology; FLAG, mouse/monoclonal (F3165), Sigma-Aldrich.

### Alkaline CIP treatment

For dephosphorylation with CIP (New England Biolab, Ipswich, MA, USA), 5–10 μl of HeLa lysate was mixed with 1× NEB buffer 3 with 0.5–1.0 unit CIP/μg protein and DEPC H_2_O in 20 μl and incubated at 37°C for 60 min. Control reactions contained all of the above except CIP and were also incubated at 37°C for 60 min.

### Northern blotting

RNA was extracted from 48 h untreated or treated HeLa cells with TRI-REAGENT (Cincinnati, OH, USA) according to the manufacturer's suggested protocol. Equal volume of gel loading buffer was added to 10–16 μg of samples and then heated at 95°C for 5 min. RNA was run on a 6% denaturing polyacrylamide gel (Invitrogen) in 1× Tris-Borate-EDTA (TBE) at 200 V for approximately 30 min. After a 10 min wash in TBE, RNA was transferred to membrane with iBlot DNA transfer stacks (Invitrogen) and iBlot Gel Transfer device (Life Technologies, Grant Island, NY, USA) using program 5 for 5 min. Membrane was rinsed in ultrapure water then dried and crosslinked using a UV cross-linker (UVP, Upland, CA, USA) at a setting of 120,000 μJ/cm^2^. The membrane was then pre-hybridized in a hybridization bottle using 15 ml of Ultrahyb Ultrasensitive Hybridization buffer (Ambion Life Technologies, Grand Island, NY, USA) for 30 min at 42°C in a rotating hybridization oven. The membrane was then probed overnight with a DIG-labeled DNA oligo probe, which hybridizes to full-length scaRNA9 and the mgU2-30 fragment, as described elsewhere (Logan et al., 2018). Membranes were then prepared for detection using the DIG Wash and Block kit (Invitrogen) following the manufacturer's suggested protocol with the Anti-DIG antibody used at 1:10,000. Detection was carried out using CSPD (Roche, Mannheim, Germany) following the manufacturer's suggested protocol. Blots were imaged using a Chemidoc imager (Bio-Rad). Adjustments to images were made using the transformation settings on QuantityOne software and applied across the entire image.

### Immunofluorescence (IF)

IF, image capture and processing were conducted as previously described (Logan et al., 2018). Briefly, cells were seeded into chambered slides and untreated or treated with etoposide (9 μM) for 48 h. Cells were then fixed with paraformaldehyde, followed by extraction with triton and blocking with normal goat serum. Anti-SMN and anti-coilin antibodies (described above) were then used, along with the appropriate secondary antibodies. DAPI was used to stain the nucleus.

### Statistical analysis

Student's *t*-test was performed to determine statistical significance, *P≤0.05.
